# Functional Bowel Disorder Management in Routine Practice with Tips for Hot Topics: Expert Opinion Review

**DOI:** 10.5152/tjg.2024.24029

**Published:** 2024-06-01

**Authors:** Filiz Akyüz, Altay Çelebi, İbrahim Doğan, Yusuf Erzin, Taylan Kav, Müjde Soytürk, Dilek Oğuz, Birol Özer, Sabahattin Kaymakoğlu

**Affiliations:** 1 Department of Gastroenterology, İstanbul University İstanbul Faculty of Medicine, İstanbul, Türkiye; 2 Department of Gastroenterology, Kocaeli University Faculty of Medicine, Kocaeli, Türkiye; 3 Private Koru Hospital, Ankara, Türkiye; 4 Department of Gastroenterology, İstanbul Cerrahpaşa University Cerrahpaşa Faculty of Medicine, İstanbul, Türkiye; 5 Department of Gastroenterology, Hacettepe University Faculty of Medicine, Ankara, Türkiye; 6 Department of Gastroenterology, Dokuz Eylül University Faculty of Medicine, İzmir, Türkiye; 7 Department of Gastroenterology, Çanakkale University Faculty of Medicine, Çanakkale, Türkiye; 8 Department of Gastroenterology, Başkent University, Adana, Türkiye

**Keywords:** Functional bowel disorders, diet, antispasmodics, gut–brain disorders, simethicone

## Abstract

Functional gastrointestinal system disorders are common problems in practice. The most common symptoms are abdominal pain, gas, bloating, diarrhea, constipation, and a mixture of these, and similar symptoms can be seen in conditions such as inflammatory bowel disease, colorectal cancer, and celiac disease depending on the age of the patient, indicating the importance of differential diagnosis. The importance of patient management is shown by making a symptom-based diagnosis and making cost-effective, that is, limited advanced examinations. The pathophysiology of irritable bowel syndrome (IBS) is multifactorial, and stress is one of the leading triggers of IBS symptoms. Therefore, terminology will change to gut–brain interaction disorders in the future, and the patient–physician relationship has a special place in the treatment of functional bowel disorder. Dietary recommendation and medical treatment in IBS should be determined according to the predominant symptom and symptom severity. In addition to diet, some lifestyle changes can also be helpful in reducing IBS symptoms. Antispasmodics and antidepressants are not fast-acting. These drugs should be used for at least 2-4 weeks to see the efficacy of treatment. Drugs should be used according to the standard recommended duration and dose in intermittent treatments.

**Main Points**

- Functional gastrointestinal disorders are seen in 40% of the population at any time in their lives and may cause recurrent chronic symptoms in approximately 2/3 of these patients. Before diagnosing FBD, detailed information about drug and operation history and dietary changes should be obtained from the patients. It was stated that the diagnosis of functional bowel disorders (FBD) was based on evaluation of the clinical symptoms and not a biomarker.
- Perceptions of physicians on FBD also play a decisive role in patient–physician relationship. Some physicians believe that FBD is a psychological disorder or a response to stress. The patient should be informed about why the test results are normal, that their symptoms can be triggered by stress, but may also occur during a stress-free period, that this disease will not shorten their life span, and that it is not associated with cancer, hence the prognosis of the disease.
- Dietary recommendation and medical treatment in irritable bowel syndrome (IBS) should be determined according to the predominant symptom and symptom severity.
- Otilonium and pinaverium have been reported to have the most evidence for efficacy among antispasmodics in IBS. In a meta-analysis that included 2585 pts showed that otilonium and the alverine/simethicone combination produced significant values in global improvement while the pinaverium/simethicone combination showed improvement in bloating.
- Neuromodulators are effective at different levels in the brain–gut axis. Therefore, we can use for all symptoms of FBD.

## Introduction

Functional bowel disorders (FBD) is common problem in our routine practice. Although we use the Rome criteria in clinical practice, some gray areas can be confusing and challenging in treatment. In this article, we evaluated the following topics in the management of FBD in real practice:

1. Diagnosis and differential diagnosis
2. Overall clinical approach, initial evaluation
3. Informing on treatment goals
4. Diet and treatment in irritable bowel syndrome (IBS)
5. Diet and treatment in functional constipation (FC)
6. Diet and treatment in functional diarrhea (FD)
7. Diet and treatment in functional abdominal bloating/distension
8. Lifestyle changes
9. Follow-up and monitoring in FBD

In PubMed/MEDLINE, the past and recent literature in English on FBD was reviewed, and each team of experts wrote their opinions on these areas. These issues were then discussed within the groups and the final version of the article were drafted.

## Diagnostic Criteria and Differential Diagnosis in Functional Bowel Disorders

### How Common Are Functional Gastrointestinal Disorders?

Functional gastrointestinal (GI) disorders are seen in 40% of the population at any time in their lives and may cause recurrent chronic symptoms in approximately 2/3 of these patients. Due to the prevalence of the disorder, delays in diagnosis and treatment cause increased expenses as a result of unnecessary hospital visits, consultations, surgical interventions, over-the-counter drug use, and most importantly, loss of workforce.^1^

## What ARE the Underlying Pathophysiology of the Disorder?

Although its pathophysiology is not fully known, it is accepted as a biopsychosocial disorder characterized by GI flora disorders (dysbiosis), 5-HT-mediated visceral hypersensitivity, and a bilateral disorder in the brain–gut interaction accompanied by impaired motility.^2^ Therefore, Rome IV will be revised to Disorders of gut–brain interactions in the future (https://theromefoundation.org/rome-iv/rome-v/).

### What Are the Usual Symptoms in Functional Bowel Disorders?

The most common symptoms are abdominal pain, gas, bloating, diarrhea, constipation-, and a mixture of these, and similar symptoms can be seen in conditions such as inflammatory bowel disease (IBD), colorectal cancer, and celiac disease depending on the age of the patient, indicating the importance of differential diagnosis.

Nowadays, treatment of the dominant symptom(s) stands out rather than revealing the underlying pathophysiological mechanisms in FBD. Due to the absence of an organic disease in FBD, patient complaints are often underestimated by physicians and patients are thought to have psychological problems. In difference due to this acceptance as a second-class disease, so to say, can put patients in a dead end. Today, the diagnosis of FBD depends on the symptom-based 2016 Rome IV criteria, and FBD is more common in women.^3^

In patients diagnosed with FBD who meet the symptom criteria, differential diagnosis should be made with cost-effective testing, in other words, diagnosis should be addressed rather than elimination.^4^ To give an example, while it is sufficient to just monitor a student who has short-term abdominal pain and diarrhea attacks before the exam, colonoscopy should definitely be performed on an elderly person with worsening similar complaints, for cost-effective differential diagnosis. The alarm symptoms that require a wider differential diagnosis are, age over 50, recent complaints, anemia, loss of appetite, weight loss, rectal bleeding, family history of colon cancer/IBD, and presence of nocturnal symptoms.^5,6^

## Why Is It Important to Take a Good History?

Before diagnosing FBD, detailed information about drug and operation history and dietary changes should be obtained from the patients. For example, patients with complete or partial pancreatic resection or history of bariatric surgery (such as a mini gastric bypass) may have symptoms similar to symptoms in IBS with predominant diarrhea. Also, frequently used beta-blockers or calcium channel blockers may mimic FC, or drugs such as metformin and proton pump inhibitors may mimic FD. In such cases, it becomes important to question the relationship between drug initiation and the appearance of symptoms. Similarly, abdominal distention may occur in individuals due to increased fiber intake who switch from a conventional diet to a vegan diet. All these examples point to the importance of taking a good history.

The differential diagnosis of diarrhea-dominated FBD includes lactose intolerance, celiac disease, non-celiac gluten sensitivity, parasitosis, IBD, microscopic colitis, pancreatic exocrine insufficiency, hyperthyroidism, bariatric neuropathy, intestinal hypomotility secondary to systemic scleroderma resulting in bacterial overgrowth syndrome. On the other hand, the differential diagnosis of constipation-dominated IBS includes strictures due to colorectal tumor, previous diverticulitis and inguinal hernia, and hypomotility due to hypothyroidism.^3^

## How ARE Functional Bowel Disorders Classified?

According to the Rome IV criteria, FBD is Classified as:^3,^^6^

- Irritable bowel syndrome [IBS with predominant constipation (IBS-C), IBS with predominant diarrhea (IBS-D), IBS with mixed bowel habits (IBS-M), IBS unclassified (IBS-U)]
- FC
- FD
- Functional abdominal bloating/distension
- Unspecified FBD
- Opioid-induced constipation

### Which Tests Should Be Considered in the Differential Diagnosis of Functional Bowel Disorders?

Diagnostic criteria and recommended tests for differential diagnosis are given in the Table 1.^3,7^

As can be seen in the Table 1, the importance of patient management is shown by making a symptom-based diagnosis and making cost-effective, that is, limited advanced examinations.

### What Is Bristol Stool Form Scale?

**Figure 1.**
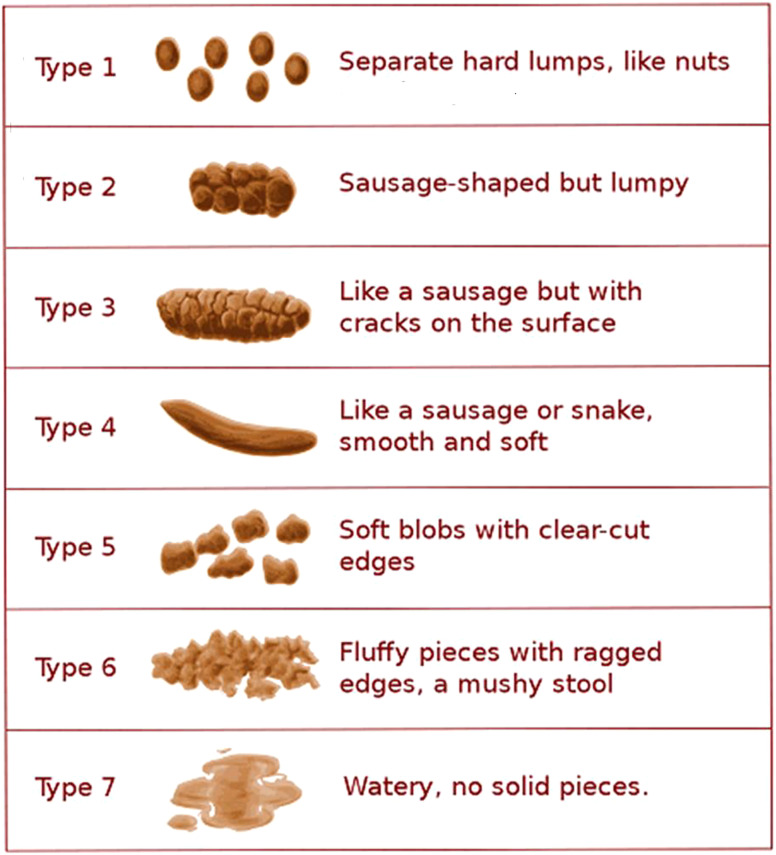
Bristol stool scale (Copyright 2000 © by Rome Foundation. All Rights Reserved.).

The Bristol stool form scale (Figure 1) can be used to help patients describe the stool shape more easily and also for standardization.^3^

## Overall Clinical Approach, Initial Evaluation

### What Is Functional Bowel Disorder?

The most common symptoms suggestive of a GI disorder are abdominal pain, diarrhea, constipation, and bloating. Organic pathologies may also cause these symptoms, we know that in a significant number of patients, no conclusive results have been achieved from all the tests performed, therefore the term “functional” disorders have been used for these symptoms. Since the ambiguous term “functional” reduces the importance of the disorder and there are many factors that play a role in the development of the disorder, this condition is called gut–brain interaction disorders. Irritable bowel syndrome, that is classified in this group of disorders, is a leading reason for patients to consult a doctor, and is accompanied by symptoms such as FC/diarrhea, or bloating.^8^

As there is no biomarker for the diagnosis of FBD, the diagnosis of these disorder is based solely on the clinical examination and patient history. A good history should be taken and the patient’s dominant symptom should be determined. Appropriate definition of a group of disorders that have a highly variable pathophysiology is crucial step to provide effective treatment. When we include the common disorders mentioned above, approximately 33 different FBDs can be differentiated according to symptoms only. Although there has been increasing criticism of the restrictive criteria, most FBDs are well established. A worldwide internet survey showed that almost 1 out of every 2 people meet the criteria for FBD.^9^

### In Which Patient Group Is It More Common?

Young and middle age establish an important risk factor as shown by the studies, while it is less common above the age of 50. Extra-intestinal symptoms such as chronic pain syndromes and fatigue are common and have a negative impact on quality of life. The history of infection in some patients is worth mentioning. An increase in risk has been reported after acute GI system infections in both IBS and functional dyspepsia. In particular, the risk of developing IBS increases 4 times. So far, no definite relationship has been demonstrated between components of microbiota and FBD subtypes.^8^

### How Should the First Clinical Course Communication with the Patient Be?

Functional bowel disorder diagnosis is not a diagnosis based on exclusion. Patients should be informed clearly about both the name and the characteristics of the disease in an understandable way by using good communication skills. Questions about the cause of the disease, its course, and how long it takes to get a response to treatment should be addressed using as clear expressions as possible. The fact that no underlying organic pathology has been defined so far (at least until now), and the functional designation of the disease should not reduce its importance neither for the patient nor for the doctor.^5,10^

### Is Functional Bowel Disorder a Psychiatric Disease?

Addressing the complaints of people diagnosed with FBD as psychiatric origin reduces the success of treatment. Considering it as the manifestation of psychological disorders makes it more difficult to learn the symptoms that bother the patients or to detect an organic disorder, if any. As patients and their relatives hear comments such as “this is a mental disorder,” “it’s in your head,” “fix your psychology,” or “go to a psychiatrist,” they suffer from this disease for a long time without getting any better, resulting in worsening of the symptoms. At the end, the patient will stop using the drugs you prescribe, or you will start to avoid spending more time or doing more tests as a doctor, thus the vicious circle will continue to get worse. Sometimes, listening and understanding the patient without giving any medication, may even be enough for the treatment of the disease.^9^

### What Is the Natural Course of the Disease?

First of all, doctors should believe that FBDs are treatable. Just talking to the patient will have a 30% probability that the patient’s complaints will go away. In some patients the complaints may regress spontaneously within a year. Two-thirds of the patients show a chronic course with recurrent complaints.^5^

### What are the Diagnostic Criteria in Functional Bowel Disorders?

It was stated that the diagnosis of FBD was based on the evaluation of the clinical symptoms and not a biomarker. Therefore, the use of symptom-based criteria (ROMA IV) required for the diagnosis of FBD will provide a clearer diagnosis. In fact, using these criteria is not very challenging, on the contrary, it provides a more appropriate approach to the patient. During the initial examination, patients should be evaluated for alarm symptoms, particularly for weight loss or rectal bleeding. It is more appropriate to evaluate according to the criteria in chronic patients who have complaints for more than 6 months.^3,6^ In addition to these criteria, information on the patient’s history will increase the success of the diagnosis, such as absence of nocturnal diarrhea, presence of depression, or extra-intestinal symptoms. A detailed evaluation of the patient’s history may reveal conditions that may trigger the disease. Since the drugs may also cause constipation or diarrhea, the drugs used by the patients should be reviewed. The absence of an abnormal finding on physical examination can be interpreted in favor of FBD. It is useful to review the patient’s previous tests before requesting one.^3^

### Which Tests Should Be Requested in the Initial Evaluation?

It should be kept in mind that the diagnostic criteria for FBD may also be met in people with organic diseases such as celiac disease or IBD, and appropriate simple tests should be performed. The most useful laboratory tests are the complete blood count and CRP levels. Apart from these, tests such as the whole biochemistry panel and thyroid function tests do not contribute much, thus, they should not be requested unless there is a very serious doubt.^7,11^

### Should Celiac Tests Be Done Routinely?

Celiac disease serology is more useful if an additional test is required. Although it is generally requested in patients with refractory complaints, it may be requested during the initial examination in patients with a predominant complaint of diarrhea and suspected FBD. Although celiac disease is seen around 1% in the community, the probability of having celiac disease in patients with IBS symptoms has increased approximately 3 times. Interestingly, this is not only true for diarrhea, but also for those with constipation. The decision should be made according to the presence of alarm symptoms in patients with new complaints.^6,11^

### Which Patients Should Undergo Endoscopic Procedures?

A patient over 50 years of age and who has never had a screening colonoscopy requires a colonoscopy before the diagnosis of IBS. Moreover; unexplained rectal bleeding and iron deficiency regardless of age, history of celiac disease, IBD or colorectal cancer in the first-degree relatives are conditions that require further investigation. In patients with diarrhea, it may be suspected to have “missed an organic disease.” Fecal calprotectin (FCP) test is sufficient to differentiate organic from functional. Roughly, if FCP is <50 µg/g, it is considered normal, if FCP is >200 µg/g, it may be IBD. It should be noted that the diagnostic contribution of colonoscopy is very low in people who meet the criteria for IBS.^8,11^

## Informing On Treatment Goals

### A Pinch of Empathy

The patient–physician relationship has a special place in the treatment of FBD. These patients often have negative thoughts that they cannot be understood by the physician and will encounter a lack of empathy. Some patients also report that the physician visits do not contribute to their treatment and knowledge/experience about the disease.^5^

### What Do Patients Know? What Do They Expect from the Doctor?

In a survey including more than a thousand patients, over 90% of patients expressed that they wanted their doctor to provide comprehensive information about IBS, provide resources for additional information, be a good listener, answer their questions, and provide information about medications. The results from the previous health experiences of these patients are as follows: 40% said that their physician provided information, 64% said that the physician listened to them, and 47% said that they felt supported. Similarly, many patients do not have sufficient knowledge of the nature and prognosis of IBS. Some patients do not know that abdominal pain is an important symptom seen in IBS, and some think that colonoscopy will help to diagnose IBS. Again, a significant portion of patients believe that IBS increases the risk of developing IBD, while others think that it may lead to cancer.^5,6^ Functional bowel disorder does not transform to cancer. Functional bowel disorder is not the precursor disease of IBD. It does not increase the risk of transform to IBD.^12^

Perceptions of physicians on FBD also play a decisive role in patient–physician relationship. Some physicians believe that FBD is a psychological disorder or a response to stress. As seen, some opinions and perspectives of both patients and doctors about FBD have the potential to negatively impact patient-physician relationship.^5^

### What are the Factors in Patient–Physician Relationship that May Positively Impact the Success of Treatment?

The following may positively impact patient-physician relationship:

- Listening to the patient carefully
- Focusing on the patient’s problems during the interview
- Making an effort to understand the patient’s feelings, showing empathy
- Informing the patient about the disease and treatment goals
- Discovering what is important to the patient and determining priorities in treatment together with the patient
- Involving family members, if possible, in the treatment so that they can positively reinforce the patient’s behavior^5^

### How Should the Treatment Goals be Explained to the Patient?

It is very important to make a clear statement about the nature of FBD. Because their complaints recur, these patients are concerned that their diagnosis is wrong and an important underlying disease is missed. The patient should be informed about why the test results are normal, that their symptoms can be triggered by stress, but may also seen in a stress-free period, that this disease will not shorten their life span and that it is not associated with cancer, hence the prognosis of the disease.^10^

## Diet and Treatment in Irritable Bowel Syndrome

### Which Factors Play a Role in Treatment Decisions in Irritable Bowel Syndrome?

Dietary recommendation and medical treatment in IBS should be determined according to the predominant symptom and symptom severity.^13^

### For Which Type of Irritable Bowel Syndrome Should the Low Fermentable Oligosaccharides, Disaccharides, Monosaccharides, and Polyols Diet Be Recommended?

Fermentable oligosaccharides, disaccharides, monosaccharides, and polyols (FODMAPs) led to increased GI water secretion and increased fermentation in the colon, thereby producing short-chain fatty acids and gases in the lumen, which can lead to bloating and trigger food-related symptoms in patients with IBS. Most studies have reported that the low FODMAP diet is beneficial for IBS symptoms, particularly abdominal pain and bloating.^14-16^ The first step in the FODMAP diet is the introduction of low-FODMAP foods. Responders to the diet can be identified within 4-6 weeks. In patients who do not respond to diet within 4-6 weeks, the diet should be discontinued. In responders, the second phase is to add restricted foods gradually to the low-FODMAP diet after the sixth week. In phase 3, the diet is individualized by avoiding foods that trigger symptoms. Implementing the low-FODMAP diet under the supervision of a dietitian helps to get a better response. If this cannot be done, the patient should be provided with descriptive documents. Low-FODMAP diet can particularly be recommended in IBS with predominant diarrhea, accompanied by gas bloating.^17^ The FODMAP diet is discussed in more detail under the section “Functional Diarrhea.”

### How Should Fiber Be Supplemented?

Soluble fibers such as psyllium (ispaghula), are effective for abdominal pain, constipation, and global symptoms in IBS. Soluble fiber is also found in oat bran, barley, and beans. Psyllium should be started with 3-4 g daily and gradually increased so as not to cause bloating. The amount of psyllium used in the studies is between 6 and 30 g/day.^18^ Insoluble fibers such as wheat bran may aggravate symptoms, as well as have a low water-holding capacity due to colonic fermentation and increase gas-bloating. Though most patients with IBS have been reported to improve with a gluten-free diet in observational studies, the results of randomized controlled studies are conflicting. Therefore, gluten-free diet is not recommended in the guidelines in IBS.^14,17^

### What Are the Drug Treatments in Irritable Bowel Syndrome and How Are They Used?

#### Antispasmodics:

Antispasmodics may be effective for general symptoms and abdominal pain in IBS. Dry mouth, visual impairment, and dizziness are common side effects.^6^ In a meta-analysis of 8 studies comparing pinaverium with placebo in patients with IBS, or of pinaverium was found to be 3.43 (2.00-5.88), and numbers need to treat (NNT) was 4. Otilonium and pinaverium have been reported to have the most evidence for efficacy among antispasmodics in IBS.^19^ Studies have shown that the superiority of antispasmodics over placebo begins at approximately 4 weeks and becomes more significant between 8 and 12 weeks. It should be recommended to be used before or during meals.^18^ The simultaneous use of more than one antispasmodic drug should not be recommended. It is necessary to use at least 4-6 weeks for the effect to be seen. In randomized, placebo-controlled studies of combination preparations of pinaverium and alverine citrate with simethicone in patients with IBS, the improvement in symptoms of abdominal pain and bloating was significantly superior to placebo.^20^ In a meta-analyses which included 2585 pts showed that otilonium and the alverine/simethicone combination produced significant values in global improvement while the pinaverium/simethicone combination showed improvement in bloating.^21^ Antispasmodics should be continued for 8-12 weeks after initiation.^5,17,18^

#### Probiotics:

They may be effective for abdominal pain and global symptoms in IBS. Although meta-analyses have reported that the efficacy of combined probiotics in IBS is better than single strains, there is significant heterogeneity and bias in the studies. Currently, there is insufficient evidence to strongly recommend probiotics for the treatment of IBS. Probiotics may be recommended to use on an individual patient-need basis in patients with diarrhea-dominant IBS (IBS-D) or mixed IBS which is accompanied by bloating and gas.^22^

#### Loperamide:

It can be effective in IBS-D. Dose increase should be gradually titrated to prevent side effects such as abdominal pain, nausea, vomiting, and constipation. Loperamide should be started with 2 mg tablets, 1 tablet twice a day, and could be increased up to a maximum of 16 mg/day, in 3-4 divided doses. Continuous use is not recommended.^6^

#### Polyethylene Glycol:

It is an effective treatment for constipation in IBS. However, randomized clinical trials (RCTs) have shown that polyethylene glycol (PEG) is insufficient in reducing general symptoms or pain in patients with IBS with predominant constipation (IBS-C). It should be considered that the effect may start late (up to 2-3 days), and the dose should be titrated according to the response. The sachet forms can be titrated up to 30 g/day, starting with a dose of 10 g/day. Abdominal pain may occur as a side effect.^6^

#### Mint Oil:

May be effective for abdominal pain and global symptoms in IBS. Gastroesophageal reflux (GER) is the most common side effect. It should be taken 30-60 minutes before meals. The clinical benefits of peppermint oil in patients with IBS are mostly attributed to L-menthol blocking calcium channels and accompanying smooth muscle relaxation. It should be noted that very few of the peppermint oil preparations used pass efficacy and safety tests. In studies, doses of 3 × 180 mg to 2 × 450 mg have been used.^23^

#### Tricyclic Antidepressants:

It is an effective treatment option for global symptoms and abdominal pain in IBS. Studies with tricyclic antidepressants (TCAs) in IBS patients found NNT to be 4.5. Patients should be informed about the purpose of use and possible side effects. Amitriptyline should be started as a single dose of 10 mg per day before bed-time at night and should gradually be titrated to a single dose of 25 mg/day, or maximum 50 mg/day, if necessary. Drowsiness and dry mouth are the most common side effects. The NNH (number needed to harm) of amitriptyline is reported to be 9. It should also be kept in mind that less frequent side effects such as urinary retention, constipation, and cardiac arrhythmias may be seen.^6,13^

#### Selective Serotonin Reuptake Inhibitors:

Selective serotonin reuptake inhibitors (SSRIs) may be a treatment option for global symptoms and abdominal pain in IBS. Selective serotonin reuptake inhibitors were not found to be as effective as TCAs in meta-analyses. Primarily, it is a treatment option to be considered in patients with IBS-C who are unresponsive to initial treatments. Patients should be informed about the purpose of use and possible side effects.^6,13,18^

#### 5-HT3 Receptor Antagonists:

They are effective second-line drugs for patients with IBS-D. Alosetron and ramosetron are not available in many countries. Ondansetron can be used as an alternative. Its dose can be titrated from 4 mg once daily to a maximum 8 mg 3 times daily. Constipation is the most common side effect of this group of drugs. Ischemic colitis has been reported as a side effect with alosetron. These drugs are probably the most effective option for patients with IBS-D and can be used in patients unresponsive to conventional therapy.^6,13^

#### Rifaximin:

Rifaximin, a luminally acting non-absorbable antibiotic, is an effective treatment option for patients with IBS-D, though it has limited efficacy in abdominal pain. The NNT of rifaximin in patients with IBS-D is 9, and the NNH is 8971. The results of a double-blind RCT have shown that, in patients who responded to the initial dose of 3 × 550 mg/day for 2 weeks, the 2-weekly treatments could be repeated 2 more times.^6,13^ Rifaximin can be used in this indication for 14 days at a dose of 3 × 400 mg/day or 2 × 550 mg/day.

#### Eluxadoline:

Eluxadoline, a mixed opioid receptor drug, is an effective second-line drug for patients with IBS-D. It was found to be significantly superior to placebo at a daily dose of 2 × 100 mg.^24^

#### Bile Acid Sequestrants, Colesevelam (1875 mg/day):

Given the lack of controlled studies of bile acid sequestrants in patients with IBS-D, the use of bile acid sequestrants is left to the discretion of the clinician in guidelines, as a group of patients may benefit from this therapy.^10^

#### Guanylate cyclase-C agonists, Linaclotide and Plecanatide:

They are an effective second-line treatment option for patients with IBS-C. Although diarrhea is a common side effect, they are the most effective secretagogues available for patients with IBS-C.^6,13^

#### Intestinal Chloride Channel Activator, Lubiprostone:

It is an effective second-line drug for patients with IBS-C. Diarrhea, as a side effect, is less prevalent than other secretagogues, but nausea is a common side effect.^6,13^

#### Sodium–Hydrogen Exchange Inhibitor, Tenapanor:

It is an effective second-line drug for patients with IBS-C. Diarrhea is a common side effect with this drug.^6,13^

#### 5-HT4 Receptor Agonists, Tegaserod and Prucalopride:

Tegaserod is an effective second-line drug for patients with IBS-C, but has been withdrawn from market due to cardiac side effects. Prucalopride is used in FC.^6,13^

### Is Pscycotherapy Effective in Irritable Bowel Syndrome?

Cognitive behavioral therapy specific to IBS may be an effective treatment option for global symptoms. Another effective treatment option for global symptoms is intestinal hypnotherapy. Psychological treatments should be considered if symptoms do not improve after 12 months of drug therapy. Psychological treatments can be beneficial at an earlier stage depending on patient preference and its availability.^6,7,13^

### How Should Treatment Be Managed in Resistant Irritable Bowel Syndrome?

Patients with severe and resistant IBS who do not benefit from current treatments should be referred to the third step to be managed with a multidisciplinary approach. In these patients, the diagnosis should be reconsidered with further targeted investigations. Opioid prescriptions should be avoided. Fecal transplant is not recommended for the treatment of IBS in the light of current evidence. The combination of gut-brain neuromodulators may be considered for more severe symptoms, with caution against the risks of serotonin syndrome.^6,18^

Medical therapies are summarized in Table 2.

## Diet and Treatment in Functional Constipation

### What Is Functional Constipation?

A patient has constipation if the frequency of defecation is less than 3 times a week, or if the patient needs excessive straining in at least 1 of the 4 defecations, if the stool is hard and lumpy, if the patient feels anorectal blockage or obstruction, if the patient feels incomplete evacuation, or if defecation is manually assisted.^12,25^

### What Are the Risks and Complications of Constipation?

- Impairment in quality of life
- Hemorrhoids
- Anal fissure
- Fecal impaction
- Hospitalization
- Increased mortality in older patients with cardiovascular disease^26^
- Pelvic floor damage

### How Should Pre-Treatment Evaluation Be Performed?

Patient history and physical examination are sufficient in most patients. A limited number of basic laboratory tests are sufficient. Further investigations such as colonoscopy are required in those with alarm symptoms.^25^

### What Are the Alarm Symptoms?

- Symptoms in patients above 50 years of age
- Hematochezia
- Weight loss (more than 10 percent in the last 6 months)
- Anemia (iron deficiency anemia unexplained by any other reason)
- Family history of colon cancer or inflammatory bowel disease
- Recurrent fever
- New onset constipation, especially in the elderly
- Worsening, exacerbating constipation
- Anorexia, nausea, vomiting
- Palpable abdominal/rectal mass
- Severe unexplored symptoms^7,25^

### How Long Should Constipation Treatment Continue?

Constipation treatment should be continued as long as the patient’s complaints continue. It is usually continuous. During recovery periods, a break can be taken. The effective drug is continued as long as its efficacy lasts. The drug can be changed or combined with drugs with different mechanisms of action in case the efficacy is insufficient.^25^

### Facts About Constipation

Dolichocolon (long colon) is not a cause of constipation. Unless there is dehydration, there is no benefit in taking more fluids than the daily requirement. Hypothyroidism can cause constipation, but hypothyroidism is rare among constipated patients. While fiber is good for some patients, it can increase symptoms in others. Increasing physical activity in the elderly may be beneficial. Normal doses of stimulant laxatives do not have a colon-destructive effect. There is no scientific basis to support the notion that stimulant laxatives are detrimental to the colon, either in animals or humans. The key takeaway for healthcare practitioners is that when used appropriately, stimulant laxatives appear to be a safe and effective option, with no potential for addiction. Tolerance does not develop to stimulant laxatives. Some patients are laxative-dependent for defecation, but this is not due to their previous use of laxatives. They need it. Laxative discontinuation does not cause rebound constipation. The use of laxatives can be abused, but not habited.^12,25^

### What Should Be the Goal of Constipation Treatment?

In the short term, the treatment goal is to improve the frequency of defecation and the hard lumpy stool, to eliminate the need for excessive straining, to eliminate the anorectal blockage or obstruction and the feeling of incomplete evacuation, and to eliminate the need for manual assistance in defecation. The dose and duration of treatment should be individualized towards this goal. The long-term goal is to prevent complications and improve quality of life.^12^

### Treatment: General Precautions and Lifestyle Changes

- Increase in physical activity
- Increase in fluid intake (1.5-2 L daily)
- Increase in dietary fiber intake (gradually increase to 20-30 g daily)
- Breakfast within 1 hour after waking up in the morning
- When the feeling of defecation occurs, it should not be postponed, especially do not miss the after-meal bowel movements
- When sitting in the toilet, the knees should be higher than the hips and leaning slightly forward is recommended
- Do not push excessively and do not stay in the toilet for more than 5 minutes^12,25^

### Treatment: Examples of Fiber Ratios in Food

### Drug Treatment

#### Osmotic Laxatives:

It is usually the first-line treatment for those who have failed fiber therapy.

- Lactulose: Fluid and electrolyte passage into the lumen provides an increase in fecal mass. It can cause bloating and cramping. Its effect starts slowly.^6,7,25^
- PEG: Efficacy for up to 6 months has been demonstrated in high quality, randomized, prospective controlled studies. It is more effective than lactulose.^5,6,25^

They can be taken as a single dose in the morning or in the evening. The dose can be gradually adjusted depending on the response

### Stimulant Laxatives

#### Bisacodyl, Sodium Picosulfate, Senna:

Mechanisms of action are reduction of intestinal fluid absorption, stimulation of intestinal motility and prostaglandin synthesis. They have a fast onset of efficacy. They can be taken in the evening. They may cause abdominal pain and diarrhea in some patients. The efficacy of bisacodyl and sodium picosulfate has been demonstrated in high-quality, randomized, prospective controlled studies.^27,28^

### Diet and Treatment in Functional Diarrhea

#### What Is the Importance of Nutrition in Functional Diarrhea?

Many patients with functional GI complaints express an increase in their symptoms with certain foods. In IBS and FD, particularly carbohydrates cause symptom worsening. Fermentable oligosaccharides, disaccharides, monosaccharides, and polyols are short-chain carbohydrates that are not fully absorbed in the small intestine. It is recommended to question the foods that cause the patient’s symptom exacerbation and to remove them from the diet first.^15,29^

### What Is the Definition of Low in Fermentable Oligosaccharides, Disaccharides, Monosaccharides, and Polyols Diet?

The restriction of oligo mono/polysaccharides is to remove fructose and lactose from the diet.

Monosaccharides: glucose, fructose, galactose, xylose, arabinose

Disaccharides: sucrose, lactose, maltose, isomaltose, trehalose

Polyol: sorbitol, mannitol, isomalt, lactitol

Oligosaccharide: maltodextrin, refined sugars, fructo-oligosaccharide, soy

### Fermentable

Oligosaccharide-containing foods, such as wheat, barley, rye, onions, leeks, garlic, artichokes, beets, fennel, peas, chicory, peanuts, cashews, lentils, chickpeas

Disaccharide-containing foods, such as milk, ice cream, yogurt, cream

Monosaccharide-containing foods, such as apple, mango, honey, high fructose corn syrup, pear, watermelon, asparagus

Polyol-containing foods, such as apple, pear, nectarine, watermelon, mushroom, flavored gums, apricot, peach, cauliflower, plum^16,30^

### Should Food or Lactose Intolerance Be Tested?

On a modified FODMAP diet, fructose, sucrose, lactose, and refined sugars can primarily remove from the diet. In particular, foods containing lactose (such as milk, dairy desserts, ice cream, etc.) should be removed from the diet first. Testing for food and lactose intolerance is not recommended. The FODMAPs can cause symptoms such as abdominal pain, diarrhea, gas and bloating.^31^

### What Kind of Fermentable Oligosaccharides, Disaccharides, Monosaccharides, and Polyols Diet Should be Recommended in Functional Diarrhea?

Meta-analyses comparing the modified FODMAP diet with various diets showed that the FODMAP diet improved global symptoms in IBS patients. These improvements have mostly been investigated in patients with IBS with predominant diarrhea (IBS-D). There are not enough studies on FD.^15^ In a recent randomized controlled trial involving IBS-D patients, the modified FODMAP diet was shown to be better than the conventional diet in improving overall GI symptom scores, stool frequency, and stool consistency at 6 weeks.^15,16,30^ A randomized clinical trial in patients with IBS in the Mediterranean region compared a modified FODMAP diet to a standard diet. Completion of the 4-week diet resulted in improved quality of life as well as symptoms in both groups. However, the modified FODMAP diet led to a greater reduction in symptoms, primarily related to abdominal pain and diarrhea.^30^ Another prospective Italian study comparing the modified FODMAP gluten-free diet and the Mediterranean diet that supports the previous one showed that the only diet that normalized stool consistency was the modified FODMAP diet.^32^ Most studies have basically evaluated the short-term (up to 4-6 weeks) effectiveness of the modified FODMAP diet. Long-term results are unclear.^11,13,16,29^ A recent randomized controlled trial involving only patients with IBS-D (IBS with predominant diarrhea) evaluated the efficacy and acceptability of the short-term FODMAP diet and the long-term modified FODMAP diet compared to the traditional diet. Both FODMAP diets have been shown to be acceptable and led to significant improvement.^15^ However, the feasibility of the FODMAP diet in the short and long term is not very practical. Sustained symptom relief with the modified FODMAP diet has been demonstrated by several long-term observational studies. Long-term restriction is not recommended due to malnutrition, difficulties in adaptation, costs and social difficulties associated with the exclusion of many nutrient-rich foods. Studies have shown that besides bifidobacteria and other microbial changes, there may be iron and calcium deficiency, which can negatively affect the health of patients.^33^ For this reason, after a short diet of 4-6 weeks, the improvement in the patient’s symptoms is observed, and a personalized FODMAP diet is recommended.^11,33^

### Should a Fat-Free Diet be Recommended?

Reducing fatty foods can also help reduce symptoms, especially in FD and IBS-D.^34^

### Should a Gluten-Free Diet Be Recommended?

Most IBS patients are on a gluten-free diet without doctor’s advice. Gluten is a complex of wheat proteins, primarily gliadins, and glutenins, commonly used as an additive in processed foods for improved texture, moisture retention, and flavor. The effect of gluten restriction in patients with IBS-D is uncertain. Studies including a small number of patients have shown that a gluten-free diet improves symptoms in IBS, diarrhea, and bloating. However, some studies have shown that patients with the HLA DQ2/DQ8 haplotype are more prominent among patients who follow a gluten-free diet and benefit from it.^35^ A recent meta-analysis on the role of a gluten-free diet in IBS failed to show benefit from a gluten-free diet.^14,36^ Two different studies have shown that any benefit of a gluten-free diet in IBS may be related to the reduction of wheat-related FODMAPs, particularly fructans, and not the gluten itself.^37^ For this reason, although a gluten-free diet is recommended for IBS-D in the 2016 ROMA IV guidelines, it is not recommended in the new guidelines due to limited data on this subject and no confirmation by new studies.^3^

### What Are the Drug Treatments in Functional Diarrhea and How Should They Be Administered?

#### Loperamide:

Antidiarrheal drugs can generally be defined as agents that reduce diarrhea symptoms by reducing stool frequency, improving stool consistency, or reducing stool weight. The best-studied antidiarrheal agents so far are loperamide and diphenoxylate. The most commonly used agent in practice is loperamide. Loperamide should be used with caution as it may cause constipation, abdominal pain and prolonged QTc when the drug is used in high doses. Also, there is no evidence that loperamide is effective in treating abdominal pain and bloating. Data on long-term use are limited. It is suitable for short-term use. Loperamide 2-4 mg (maximum 16 mg) can be used daily.^33^

#### Rifaximin:

Rifaximin is an FDA-approved, nonabsorbable antibiotic. The possible mechanism of action changes the flora, reducing the diversity in the microbiota. Colonic fermentation is reduced. Flora also changes in the ileum; this prevents mucosal inflammation, alters permeability, and reduces hyperalgesia.^38^ It is superior to placebo in improvement of general symptoms and gas and bloating in IBS-D. The same efficacy was obtained with repeated use.^39^ It is also recommended in IBS-D in the American and European guidelines. It is appropriate to use rifaximin 800-1200 mg/day for 14 days.^7^

#### Probiotics:

A meta-analysis on the efficacy of probiotics in IBS showed that they can improve general symptoms as well as some specific symptoms. On the other hand, there are studies showing that specific probiotics help reduce general symptoms in IBS-D patients, but do not improve diarrhea.^40^ Different results have been obtained in different studies. Studies were heterogeneous due to factors such as using different probiotic strains, formulations or mixtures in the studies, inclusion criteria, and comorbidity (e.g., anxiety and depression). Probiotics may improve symptoms in IBS-D and FD.^41^ Currently, stool microbiota analyses are not recommended in patients with functional bowel disease.^7^

#### Others:

Eluxodaline is recommended in IBS-D.^24^ Cholestyramine which is a bile acid sequestrant can be used in chronic diarrhea of unknown cause. The recommended dose of cholestyramine is 9 g/day.^6^ Ondansetron (5-HT3 antagonist) may also be effective in IBS-D. The daily dose is recommended as 3 × 4-8 mg.^6,13^

Medical therapies are summarized in Table 3.

### Diet and Treatment in Functional Abdominal Bloating/Distension

Functional abdominal bloating–distension is a clinical picture that is frequently confused with and overlaps with other functional digestive tract diseases. The following criteria are required for the diagnosis of functional abdominal bloating–distension according to the Rome IV criteria.^3,42^

#### Diagnostic Criteria

Abdominal bloating–distension complaints must have started 6 months ago and must meet the criteria for the last 3 months. There should be recurrent abdominal bloating and/or distension occurring at least 1 day a week, and the bloating–distension should be more dominant than the other accompanying complaints of the patient. Mild abdominal pain associated with bloating may also be present, along with minor abnormalities in defecation pattern. Existing complaints are not sufficient for the diagnosis of IBS, FD, FC, and postprandial distress syndrome. Therefore, together with the complaints meeting the above criteria, in cases where organic pathologies in the differential diagnosis are excluded, the diagnosis of functional gas/bloating is made (Table 4).^43-45^

Exclusion of organic pathologies is the most essential for differential diagnosis is. As a first step, the presence of alarm signs and symptoms should be investigated, and if present, organic pathologies should be investigated with proper examinations.^45^

#### Alarm Symptoms and Findings in a Patient with Abdominal Bloating–Distension

Anemia, abnormal liver function tests, hematochezia, involuntary weight loss, persistent nausea–vomiting, family history of GI–gynecological malignancy should evaluate carefully.^43,46^

#### Pathogenesis

**Figure 2.**
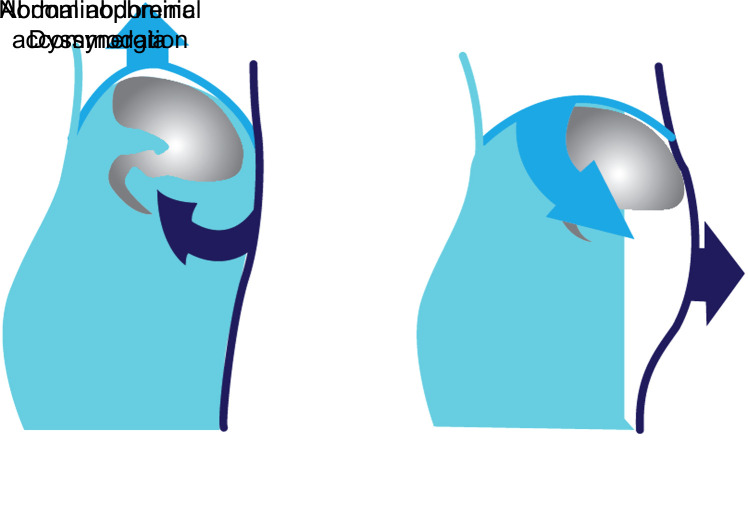
Viscerosomatic reflex.

Bloating is the subjective feeling of abdominal fullness, pressure, and gas. Distension is the objective increase in abdominal circumference. These 2 phenomena often coexist, bloating occurs without distension less than half the time. Occasional abdominal bloating–distension is very common in the general population, with a prevalence of up to 40%. Three basic pathophysiological mechanisms underlying the chronic abdominal bloating–distension. These are increased intestinal wall tension due to increased luminal gas and other content, increased perception of intestinal wall tension, and abnormal viscerosomatic reflex (abdominophrenic dyssynergia). Normally, in the presence of increased intraluminal gas, the diaphragm relaxes and displaces toward the thorax and expands the abdominal area. In some people, the diaphragm paradoxically contracts and descends into the abdominal cavity, the anterior abdominal wall relaxes and moves forward (Figure 2).^42,44,45^

The abnormal viscerosomatic reflex, which occurs as a result of brain–gut axis disorders, leads to functional abdominal bloating–distension.^42^

#### Diets That Trigger Complaints

Abdominal bloating–distension is triggered by certain dietary features. Carbohydrate intolerance is very common, the frequency of lactose intolerance is reported to be around 50%-70%. Foods containing fructose, foods rich in complex carbohydrates (potatoes, barley, oats, beans, etc.), non-absorbable sugars and synthetic sweeteners can cause gas, bloating and distension. For people with celiac and non-celiac gluten sensitivity, cereals (wheat, barley, rye, oats), fructans (cereals, pulses, peanuts, onions, garlic, cabbage, spinach, beets, peas, watermelon, grapefruit, nectarines, bananas, plums, etc.), milk, and dairy products also cause complaints of gas, bloating, and distension.^44,45^

#### Diagnostic Approach

**Figure 3.**
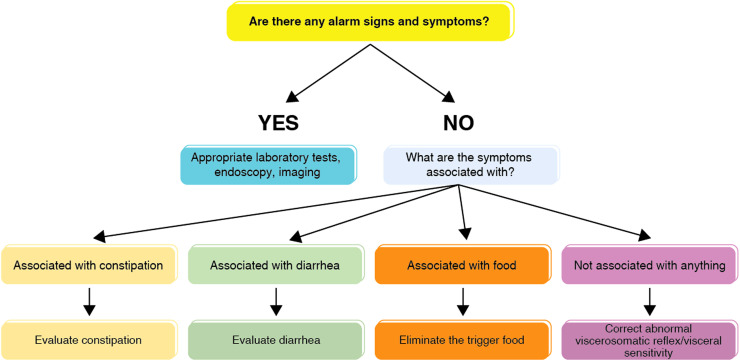
Approach for abdominal bloating/distention.

Blood count, iron parameters, vitamin B12, folic acid, celiac screening tests are routinely requested in all patients. Hydrogen respiration test is requested for carbohydrate malabsorption. An evaluation is made in the order^43,45^ shown in Figure 3.

The outlines of the treatment are as follows:

#### Reducing Intestinal Gas Formation

Dietary modifications: A low-FODMAP and lactose-free diet is comforting in a significant proportion of patients. The benefits of a gluten-free diet are not clear.

Exercise: Physical activity improves intestinal gas clearance.

Modulation of microbiota: Inconsistent results were obtained with probiotics and prebiotics. Rifaximin, a non-absorbable antibiotic, has been found to be moderately effective and can be reused when symptoms recur. Rifaximin is more effective in the subgroup with possible intestinal bacterial overgrowth.

Gas-reducing drugs: The combination of simethicone with the antispasmodic otilonium bromide or pinaverium bromide may be more effective than monotherapy. Simethicone and activated charcoal are also commonly used for this purpose.

Prokinetics: Prokinetic agents such as prucaloprid and tegaserod are effective in symptomatic relief.

Secretagogues: Drugs such as lubiprostone, plecanatide, and linaclatide have been found to be quite effective in reducing bloating in constipated patients.^44,45^

#### Reducing Visceral Hypersensitivity

Antispasmodics: Intestinal antispasmodic agents are smooth muscle relaxants that reduce the perception of intestinal wall distension. They are effective in controlling complaints of abdominal pain and bloating. Pinaverium bromide and otilonium bromide are among the most effective antispasmodics.

Neuromodulators: Drugs that are effective at different levels in the brain–gut axis include amitriptyline, imipramine, SSRIs, serotonin–norepinephrine reuptake inhibitors, levosulpiride, gabapentin, and pregabalin. Amitriptyline can be used in patients without constipation.^45^

#### Correcting the Abnormal Viscerosomatic Reflex

Biofeedback: Abdominophrenic biofeedback effectively reduces diaphragmatic and intercostal contractions, thereby improving abdominal bloating and distension. Therefore, patients should learn to do diaphragmatic breathing.^42^

Abdominal bloating–distension is the most common complaint in the population with functional GI disease. When evaluated, the patients may be diagnosed with functional abdominal bloating–distension or their complaints are a symptom complex that complements other clinical pictures. The algorithmic diagnostic approach starts with the exclusion of organic etiologies, followed by low-FODMAP diet, simethicone and antispasmodics, and other pharmacological agents used depending on the accompanying defecation disorder.^42,45^

### Lifestyle Changes

In addition to diet, some lifestyle changes can also be helpful in reducing IBS symptoms.

#### Is Exercise Beneficial in Functional Bowel Disorders?

Exercise is known to produce positive impact both physiologically and mentally. It can accelerate GI passage in patients with IBS, increase intestinal gas clearance in patients with bloating, and positively affect symptoms through the brain–gut axis. Physical activity should be offered to patients, as various exercise options, including aerobic exercise, yoga, and mountaineering, have the potential to reduce symptoms in patients with IBS.^5,33^

#### Do Stress Management and Psychotherapy Matter?

The pathophysiology of IBS is multifactorial and stress is one of the leading triggers of IBS symptoms. In turn, symptoms also trigger stress, which can ultimately become a difficult cycle to break. Although the level of evidence is not high, activities such as meditation, yoga and breathing exercises that the patient will do regularly can help prevent and relieve symptoms. Another stress-reducing approach is for patients to spare time for themselves and relax. The impact of this approach on symptoms and quality of life is unclear, but it has the potential to be beneficial when considering the stress-IBS relationship. Psychotherapies, including gut-directed cognitive behavioral therapy and bowel-directed hypnotherapy, target cognitive and emotional factors that influence the appearance, perception, and interpretation of symptoms and reduce the severity of IBS symptoms. Some of the major cognitive emotional factors that affect symptoms in IBS are: fear of symptoms, perceiving pain as a disaster, increased awareness of symptoms, somatization, and stress sensitivity. The frequency of anxiety and/or depressive disorders is high in patients with IBS. Treating underlying anxiety and depression can help manage IBS symptoms. Therefore, patients should be referred to a psychiatrist at an early stage, so that, the clinical course of IBS can be changed and the symptoms can be prevented from becoming resistant.^5,10,33^

#### Do Alternative Treatments Matter?

There are publications showing that acupuncture can regulate the brain–gut axis and improve visceral hypersensitivity. In particular, electro-acupuncture can show a therapeutic benefit on abdominal pain and bloating in IBS with predominant constipation. It can also positively impact defecation frequency, constipation, defecation difficulty, and other main symptoms. Electro-acupuncture can also help relieve depression, anxiety, and other psychological symptoms. Due to the low level of evidence, acupuncture, bioresonance and ozone therapy are not routinely recommended in patients with IBS.^33^

## Follow-Up and Monitoring in Functional Bowel Disorders

### How Often Should the Patient Be Followed Up?

Functional diseases are chronic in nature. The average recurrence rate after treatment is around 50%. Spontaneous regression of symptoms may be observed in some patients during follow-up. Follow up is important; however, there is no clear data on how long the follow-up will be. If symptoms change, different symptoms appear, or the severity of the symptoms persist, then the diagnosis should be reconsidered.^5,8,10^ Treatment is symptomatic and usually lasts 12 weeks. If responsive, 61% of patients respond in 6 weeks. Treatment should be continued at least for 4 weeks in order to see a treatment response.^9^

### How Is the Duration of Treatment Determined?

The duration of treatment varies according to the type of the disease (symptom character) and the drug used.^3,6,7,11,13^

- Treatments as needed (such as loperamide)
- Long-term treatments that last >6 months (antidepressants)
- Short-term treatments that last 4 weeks
- Generally accepted treatment duration is 8 to 12 weeks
- Patients should be invited for a follow-up visit after week 4
- If the patient is responsive, intermittent treatment with drugs that benefit the patient is safer. If the complaints recur, repeating the treatment 2-3 times a year for 3-6 months is appropriate.
- If the patient is unresponsive, it is important to perform the necessary tests and evaluate the alarm findings.

**Figure 4.**
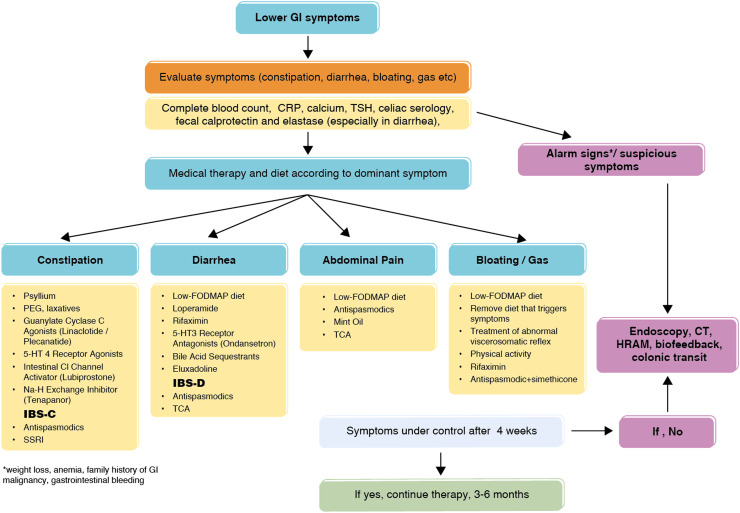
Management of functional bowel disorders.

Antispasmodics and antidepressants are not fast-acting. These drugs should be used for at least 2-4 weeks to see the efficacy of treatment. Drugs should be used according to the standard recommended duration and dose in intermittent treatments. Management of functional bowel disorders summarized in Figure 4.

## Figures and Tables

**Table 1.** Differential Diagnosis of Functional Bowel Disorders

**Disease**	**Diagnostic Criteria**	**Differential Diagnostic Tests**
IBS	Abdominal pain that started at least 6 months ago and has occurred at least once a week in the last 3 months and at least 2 of the following criteria: Association between pain and defecation Association between pain and stool frequency Association between pain and stool form	Complete blood count, CRP, thyroid hormones, celiac tests and fecal calprotectin in case of diarrhea, colonoscopy if age is >50 and/or alarm symptoms are present
Functional constipation	Difficult, infrequent defecation, and feeling of being unable to evacuate. Since the predominant complaint is not abdominal pain, it does not meet the criteria for IBS, even in case of gas and bloating.	Complete blood count, thyroid hormones, calcium, colonoscopy if age is >50, and/or alarm symptoms are present
Functional diarrhea	Recurrent soft, watery stools. Since the predominant complaint is not abdominal pain, it does not meet the criteria for IBS, even in case of gas and bloating.	Complete blood count, CRP, thyroid hormones, celiac tests, fecal calprotectin, elastase, colonoscopy if age is >50, and/or alarm symptoms are present
Functional abdominal bloating/distension	Subjective symptoms such as abdominal fullness, pressure, flatulence; conditions where there are symptoms such as objective increase in abdominal circumference (distention), mild abdominal pain, and defecation changes, but where the FBD criteria are not met.	Complete blood count, celiac tests

CRP, C-reactive protein; FBD, functional bowel disorders; IBS, irritable bowel syndrome.

**Table 2.** Medical Therapy in Irritable Bowel Syndrome (Modified from reference^10^)

**IBS with Predominant Constipation (IBS-C)**	**IBS with Predominant Diarrhea (IBS-D)**	**Abdominal Pain**
Psyllium	Low-FODMAP diet	Low-FODMAP diet
PEG, laxatives	Loperamide	
Antispasmodics	Antispasmodics	Antispasmodics Mint oil
SSRI	TCA	TCA
	Rifaximin	
Guanylate cyclase C agonists(linaclotide/plecanatide)	5-HT3 receptor antagonists (ondansetron)	
5-HT 4 receptor agonists	Bile acid sequestrants	
Intestinal Cl channel activator (lubiprostone)	Eluxadoline	
Na-H exchange inhibitor(tenapanor)		

Psychotherapy: cognitive behavioral therapy/gut hypnotherapy.

IBS, irritable bowel syndrome; FODMAP, fermentable oligosaccharides, disaccharides, monosaccharides, and polyols; PEG, polyethylene glycol; SSRI, selective serotonin reuptake inhibitor; TCA, tricyclic antidepressants.

**Table:** 

Tomato (1 medium)	1.8 g
Apricots (3 medium-sized, fresh)	1.8 g
Apple (with peel, medium size)	3.5 g
Pear (with peel, medium size)	4.6 g
Spinach (1 plate)	4.1 g
Green peas (1 plate)	7.2 g
Oat bran (28 g)	8.3 g
Plums (11 prunes)	11.9 g
Wheat bran (28 g)	12.4 g
Dry beans (1 plate)	18.6 g

**Table 3.** Medical Therapy in Functional Diarrhea

Opioid agonists Diet Bile acid sequestrants Antibiotic Selective serotonin 5-HT3 receptor antagonist	Loperamide 2-4 mg (maximum: 16 mg) Modified FODMAP diet Cholestyramine (9 g/day) Colestipol (2 g 2 x 1) Rifaximin 800-1200 mg/day for 14 days Ondansetron (4-8 mg 3 × 1)

**Table 4.** Etiologies of Abdominal Bloating/Distension

Organic Etiologies^43,44^
Bacterial overgrowth in the small bowel Carbohydrate intolerance (lactose, fructose, etc.) Celiac disease Pancreatic insufficiency Previous abdominal surgery (fundoplication, bariatric surgery, intestinal surgery, etc.) Gastric outlet obstruction Gastroparesis Ascites Gastrointestinal/gynecological malignancies Hypothyroidism Obesity Diverticular disease Chronic intestinal pseudo-obstruction
Functional Etiologies^42,44,45^
Gastric belching disorders, eating disorders (bulimia, anorexia) Irritable bowel syndrome Functional diarrhea Functional dyspepsia–postprandial distress syndrome Functional constipation, constipation due to pelvic floor dysfunction

## References

[1] Sperber AD Bangdiwala SI Drossman DA , et al. Worldwide prevalence and burden of functional gastrointestinal disorders, results of Rome foundation global study. Gastroenterology. 2021;160(1):99 114.e3. (10.1053/j.gastro.2020.04.014) 32294476

[2] Bradley S Alderson S Ford AC Foy R . General practitioners’ perceptions of irritable bowel syndrome: a Q-methodological study. Fam Pract. 2018;35(1):74 79. (10.1093/fampra/cmx053) 28985313

[3] Drossman DA . Rome IV journal articles. Gastroenterology. 2016;150(6):1262 1279.e2. (10.1053/j.gastro.2016.02.032) 27147121

[4] Ruddy J . From pretending to truly being OK: A journey from illness to health with postinfection irritable bowel syndrome: the Patient’s perspective. Gastroenterology. 2018;155(6):1666 1669. (10.1053/j.gastro.2018.11.003) 30414872

[5] Black CJ Ford AC . Best management of irritable bowel syndrome. Frontline Gastroenterol. 2021;12(4):303 315. (10.1136/flgastro-2019-101298) 34249316 PMC8231425

[6] Ford AC Sperber AD Corsetti M Camilleri M . Irritable bowel syndrome. Lancet. 2020;396(10263):1675 1688. (10.1016/S0140-6736(20)31548-8) 33049223

[7] Lacy BE Pimentel M Brenner DM , et al. ACG clinical guideline: management of irritable bowel syndrome. Am J Gastroenterol. 2021;116(1):17 44. (10.14309/ajg.0000000000001036) 33315591

[8] Linedale EC Mikocka-Walus A Vincent AD Gibson PR Andrews JM . Performance of an algorithm-based approach to the diagnosis and management of functional gastrointestinal disorders: A pilot trial. Neurogastroenterol Motil. 2018;30(1) (10.1111/nmo.13243) 29094806

[9] Irvine EJ Tack J Crowell MD , et al. Design of treatment trials for functional gastrointestinal disorders. Gastroenterology. 2016;150(6):1469 1480.e1. (10.1053/j.gastro.2016.02.010) 27147123

[10] Alammar N Stein E . Irritable bowel syndrome: what treatments really work. Med Clin North Am. 2019;103(1):137 152. (10.1016/j.mcna.2018.08.006) 30466670

[11] Moayyedi P Andrews CN MacQueen G , et al. Canadian Association of Gastroenterology clinical practice guideline for the management of irritable bowel syndrome (IBS). J Can Assoc Gastroenterol. 2019;2(1):6 29. (10.1093/jcag/gwy071) 31294724 PMC6507291

[12] Müller-Lissner SA Kamm MA Scarpignato C Wald A . Myths and misconceptions about chronic constipation. Am J Gastroenterol. 2005;100(1):232 242. (10.1111/j.1572-0241.2005.40885.x) 15654804

[13] Vasant DH Paine PA Black CJ , et al. British Society of Gastroenterology guidelines on the management of irritable bowel syndrome. Gut. 2021;70(7):1214 1240. (10.1136/gutjnl-2021-324598) 33903147

[14] Dionne J Ford AC Yuan Y , et al. A systematic review and meta-analysis evaluating the efficacy of a gluten-free diet and a low FODMAPs diet in treating symptoms of irritable bowel syndrome. Am J Gastroenterol. 2018;113(9):1290 1300. (10.1038/s41395-018-0195-4) 30046155

[15] Goyal O Batta S Nohria S , et al. Low fermentable oligosaccharide, disaccharide, monosaccharide, and polyol diet in patients with diarrhea-predominant irritable bowel syndrome: A prospective, randomized trial. J Gastroenterol Hepatol. 2021;36(8):2107 2115. (10.1111/jgh.15410) 33464683

[16] Whelan K Martin LD Staudacher HM Lomer MCE . The low FODMAP diet in the management of irritable bowel syndrome: an evidence-based review of FODMAP restriction, reintroduction and personalisation in clinical practice. J Hum Nutr Diet. 2018;31(2):239 255. (10.1111/jhn.12530) 29336079

[17] Chey WD Hashash JG Manning L Chang L . AGA clinical practice update on the role of diet in irritable bowel syndrome: expert Review [review]. Gastroenterology. 2022;162(6):1737 1745.e5. (10.1053/j.gastro.2021.12.248) 35337654

[18] Black CJ Yuan Y Selinger CP , et al. Efficacy of soluble fibre, antispasmodic drugs, and gut-brain neuromodulators in irritable bowel syndrome: a systematic review and network meta-analysis. Lancet Gastroenterol Hepatol. 2020;5(2):117 131. (10.1016/S2468-1253(19)30324-3) 31859183

[19] Bor S Lehert P Chalbaud A Tack J . Efficacy of pinaverium bromide in the treatment of irritable bowel syndrome: a systematic review and meta-analysis. Therap Adv Gastroenterol. 2021;14:17562848211033740. (10.1177/17562848211033740) PMC8447090 34539813

[20] Schmulson MJ Chiu-Ugalde J Sáez-Ríos A , et al. Efficacy of the Combination of pinaverium bromide 100 mg Plus Simethicone 300 mg in Abdominal Pain and Bloating in irritable bowel syndrome: A Randomized, Placebo-controlled Trial. J Clin Gastroenterol. 2020;54(4):e30 e39. (10.1097/MCG.0000000000001242) 31385885 PMC7069394

[21] Martínez-Vázquez MA Vázquez-Elizondo G González-González JA Gutiérrez-Udave R Maldonado-Garza HJ Bosques-Padilla FJ . Effect of antispasmodic agents, alone or in combination, in the treatment of irritable bowel syndrome: systematic review and meta-analysis. Rev Gastroenterol Mex. 2012;77(2):82 90. (10.1016/j.rgmx.2012.04.002) 22672854

[22] Ford AC Harris LA Lacy BE Quigley EMM Moayyedi P . Systematic review with meta-analysis: the efficacy of prebiotics, probiotics, Synbiotics and antibiotics in irritable bowel syndrome. Aliment Pharmacol Ther. 2018;48(10):1044 1060. (10.1111/apt.15001) 30294792

[23] Ford AC Talley NJ Spiegel BM , et al. Effect of fibre, antispasmodics, and peppermint oil in the treatment of irritable bowel syndrome: systematic review and meta-analysis. BMJ. 2008;337:a2313. (10.1136/bmj.a2313) 19008265 PMC2583392

[24] Lembo AJ Lacy BE Zuckerman MJ , et al. Eluxadoline for irritable bowel syndrome with diarrhea. N Engl J Med. 2016;374(3):242 253. (10.1056/NEJMoa1505180) 26789872

[25] Mearin F Ciriza C Mínguez M , et al. Clinical Practice Guideline: irritable bowel syndrome with constipation and functional constipation in the adult. Rev Esp Enferm Dig. 2016;108(6):332 363. (10.17235/reed.2016.4389/2016) 27230827

[26] Honkura K Tomata Y Sugiyama K , et al. Defecation frequency and cardiovascular disease mortality in Japan: the Ohsaki cohort study. Atherosclerosis. 2016;246:251 256. (10.1016/j.atherosclerosis.2016.01.007) 26812003

[27] Hinkel U Schuijt C Erckenbrecht JF . OTC laxative use of sodium picosulfate â results of a pharmacy-based patient survey (cohort study). Int J Clin Pharmacol Ther. 2008;46(2):89 95. (10.5414/cpp46089) 18218289

[28] Mueller-Lissner S Kamm MA Wald A , et al. Multicenter, 4-week, double-blind, randomized, placebo-controlled trial of sodium picosulfate in patients with chronic constipation. Am J Gastroenterol. 2010;105(4):897 903. (10.1038/ajg.2010.41) 20179697

[29] Zahedi MJ Behrouz V Azimi M . Low fermentable oligo-di-mono-saccharides and polyols diet versus general dietary advice in patients with diarrhea-predominant irritable bowel syndrome: A randomized controlled trial. J Gastroenterol Hepatol. 2018;33(6):1192 1199. (10.1111/jgh.14051) 29159993

[30] Guerreiro MM Santos Z Carolino E , et al. Effectiveness of two dietary approaches on the quality of life and gastrointestinal symptoms of individuals with irritable bowel syndrome. J Clin Med. 2020;9(1) (10.3390/jcm9010125) PMC7019629 31906563

[31] Rej A Sanders DS Buckle RL Trott N Aziz I Shaw CC . What is the optimal FODMAP threshold in IBS? J Gastroenterol Hepatol. 2021;36(6):1723 1725. (10.1111/jgh.15470) 33624857

[32] Paduano D Cingolani A Tanda E Usai P . Effect of three diets (low-FODMAP, gluten-free and balanced) on irritable bowel syndrome symptoms and health-related quality of life. Nutrients. 2019;11(7) (10.3390/nu11071566) PMC6683324 31336747

[33] Rawla P Sunkara T Raj JP . Updated review of current pharmacological and non-pharmacological management of irritable bowel syndrome. Life Sci. 2018;212:176 181. (10.1016/j.lfs.2018.10.001) 30290187

[34] Feinle-Bisset C Azpiroz F . Dietary lipids and functional gastrointestinal disorders. Am J Gastroenterol. 2013;108(5):737 747. (10.1038/ajg.2013.76) 23567355

[35] Aziz I Trott N Briggs R North JR Hadjivassiliou M Sanders DS . Efficacy of a gluten-free diet in subjects with irritable bowel syndrome-diarrhea unaware of their HLA-DQ2/8 genotype. Clin Gastroenterol Hepatol. 2016;14(5):696 703.e1. (10.1016/j.cgh.2015.12.031) 26748221

[36] Biesiekierski JR Peters SL Newnham ED Rosella O Muir JG Gibson PR . No effects of gluten in patients with self-reported non-celiac gluten sensitivity after dietary reduction of fermentable, poorly absorbed, short-chain carbohydrates. Gastroenterology. 2013;145(2):320 8.e1 -3. (10.1053/j.gastro.2013.04.051) 23648697

[37] Skodje GI Sarna VK Minelle IH , et al. Fructan, rather than gluten, induces symptoms in patients with self-reported non-celiac gluten sensitivity. Gastroenterology. 2018;154(3):529 539.e2. (10.1053/j.gastro.2017.10.040) 29102613

[38] Xu D Gao J Gillilland M 3rd , et al. Rifaximin alters intestinal bacteria and prevents stress-induced gut inflammation and visceral hyperalgesia in rats. Gastroenterology. 2014;146(2):484 96.e4. (10.1053/j.gastro.2013.10.026) 24161699 PMC3939606

[39] Menees SB Maneerattannaporn M Kim HM Chey WD . The efficacy and safety of Rifaximin for the irritable bowel syndrome: a systematic review and meta-analysis. Am J Gastroenterol. 2012;107(1):28 35; quiz 36. (10.1038/ajg.2011.355) 22045120

[40] Hungin APS Mitchell CR Whorwell P , et al. Systematic review: probiotics in the management of lower gastrointestinal symptoms - an updated evidence-based international consensus. Aliment Pharmacol Ther. 2018;47(8):1054 1070. (10.1111/apt.14539) 29460487 PMC5900870

[41] Mearin F Lacy BE Chang L , et al. Bowel disorders. Gastroenterology. 2016. (10.1053/j.gastro.2016.02.031) 27144627

[42] Damianos JA Tomar SK Azpiroz F Barba E . Abdominophrenic dyssynergia: A narrative review. Am J Gastroenterol. 2023;118(1):41 45. (10.14309/ajg.0000000000002044) 36191283 PMC9810002

[43] Mari A Abu Backer F Mahamid M , et al. Bloating and abdominal distension: clinical approach and management. Adv Ther. 2019;36(5):1075 1084. (10.1007/s12325-019-00924-7) 30879252 PMC6824367

[44] Lacy BE Cangemi DJ . A pragmatic approach to the evaluation and treatment of abdominal bloating. Am J Gastroenterol. ACG. 2022;117(5):701 705. (10.14309/ajg.0000000000001665) 35103019

[45] Lacy BE Cangemi D Vazquez-Roque M . Management of chronic abdominal distension and bloating. Clin Gastroenterol Hepatol. 2021;19(2):219 231.e1. (10.1016/j.cgh.2020.03.056) 32246999

[46] Serra J . Management of bloating. Neurogastroenterol Motil. 2022;34(3):e14333. (10.1111/nmo.14333) 35143108

